# Supplementary material for: The interactome of intact mitochondria by cross-linking mass spectrometry provides evidence for coexisting respiratory supercomplexes
Source: Mol Cell Proteomics. 2017 Dec 8;17(2):216–32. doi: 10.1074/mcp.RA117.000470 (PMC5795388; doi:10.1074/mcp.RA117.000470)
Supplement: Supplemental Data [file supp_RA117.000470_133922_0_supp_23978_fzffwf.zip › spectra_annotation/mito_DR_spectra_annotation/283-1-65-1-15-1.pdf]

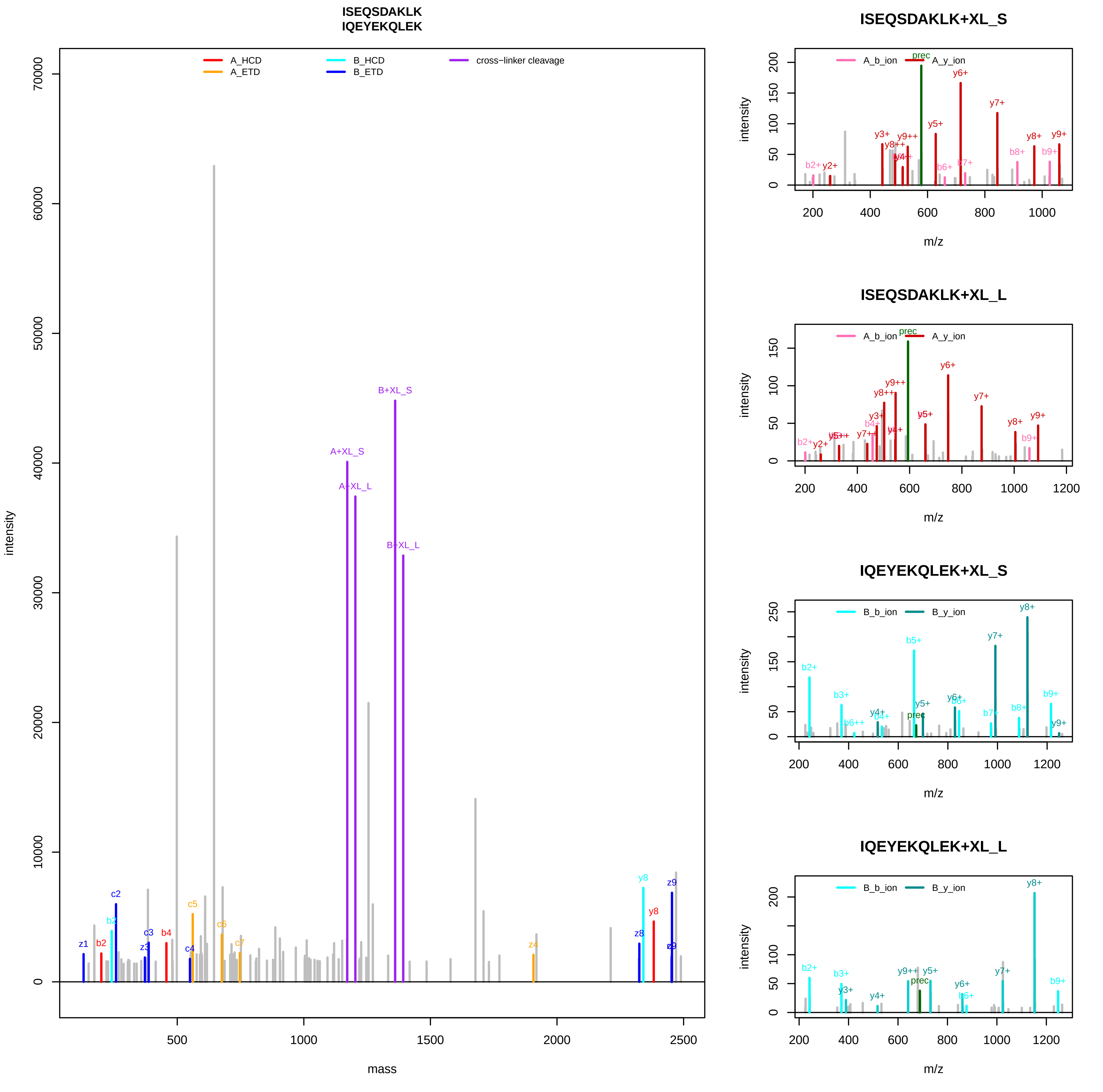
ISEQSDAKLK
IQEYEKQLEK
A_HCD
A_ETD
B_HCD
B_ETD
cross−linker cleavage
A+XL_S
A+XL_L
B+XL_S
B+XL_L
z1 b2 b2 c2 z3 c3 b4 c4 c5 c6 c7 z4 z8 y8 y8 z9 z9
intensity
mass
0 10000 20000 30000 40000 50000 60000 70000
500 1000 1500 2000 2500
ISEQSDAKLK+XL_S
A_b_ion
A_y_ion
prec
y6+ y7+ y5+ y3+ y9++ y8++ y4++ y8+ y9+ b2+ y2+ b6+ b7+ b8+ b9+
ISEQSDAKLK+XL_L
A_b_ion
A_y_ion
prec
y6+ y9++ y8++ y7+ y3+ y5+ b4+ y5++ y7++ y4+ y8+ y9+ b2+ y2+ b9+
IQEYEKQLEK+XL_S
B_b_ion
B_y_ion
y8+ y7+ b5+ b2+ b3+ b6++ y4+ b4+ y5+ prec y6+ b6+ b7+ b8+ b9+ y9+
IQEYEKQLEK+XL_L
B_b_ion
B_y_ion
y8+ b2+ b3+ y3+ y4+ y9++ y5+ prec y6+ b6+ y7+ b9+
intensity
m/z
